# On the Path toward Classifying Hormones of the Vasoinhibin-Family

**DOI:** 10.3389/fendo.2015.00016

**Published:** 2015-02-10

**Authors:** Jakob Triebel, Thomas Bertsch, Gonzalo Martínez de la Escalera, Carmen Clapp

**Affiliations:** ^1^Institute for Clinical Chemistry, Laboratory Medicine and Transfusion Medicine, Paracelsus Medical University, Nuremberg, Germany; ^2^Instituto de Neurobiología, Universidad Nacional Autónoma de México (UNAM), Campus UNAM-Juriquilla, Querétaro, México

**Keywords:** vasoinhibins, 16K PRL, classification, FASEB SRC, prolactin

Prolactin and growth hormone are produced in the anterior pituitary gland and secreted into the circulation to execute their diverse physiological effects. They are also produced at various extrapituitary sites. Apart from the traditional, well-known effects mediated by the full-length hormones, they serve as the source for vasoinhibins, which are generated by proteolytic cleavage of prolactin and growth hormone, and also of placental lactogen (1, 2). Prominent, name-giving biological effects of vasoinhibins include inhibition of angiogenesis, vasodilation, and vasopermeability (1–3). As this set of vascular effects is unique, and entirely different from the characteristics of their precursors, vasoinhibins constitute a separate hormonal family (1, 2). Apart from physiological functions, vasoinhibins seem to be involved in the pathogenesis of diabetic complications (4, 5), cancer (6, 7), and pregnancy-associated diseases (8–10). At present, approximately one and a half dozen proteins have been ascribed to belong to the vasoinhibin-family. Each of them differs by precursor, the enzyme responsible for proteolytic generation of the respective vasoinhibin-isoform, and consequently by amino acid sequence and molecular mass. The generation of vasoinhibins has thoroughly been demonstrated *in vitro*, and ongoing research has led to accumulating information about their generation *in vivo* and their pathophysiological role and clinical significance in the aforementioned diseases. However, as more information surfaced, the need for a precise terminology designating a specific vasoinhibin-isoform under study rose in parallel. For example, the term “16K PRL,” often used to describe 16 kDa-like prolactin-derived vasoinhibins, does not discriminate between the different vasoinhibin-isoforms present. Of note, prolactin-derived vasoinhibins alone include several proteins with <4 kDa difference in molecular mass between 14 and 18 kDa. This is of relevance as it remains to be shown whether and to what extent each of the different proteases contributes to the physiological release of vasoinhibins and how the generation of vasoinhibins is modified under disease conditions. Accordingly, the total composition of endogenous vasoinhibins in the circulation or at the target-tissue level has yet to be determined. To address the need for a terminology with which it is possible to precisely differentiate between proteins ascribed to the vasoinhibin-family, Vazquez Rodriguez et al. proposed a classification according to precursor, proteolytic enzyme involved in the generation of the respective protein, its sequence and theoretical molecular mass, considering a variety of 20 proteins derived from prolactin, growth hormone, and placental lactogen (11). However, because of the presence of important limitations in understanding and a number of unresolved issues, which are presently inherent to the field, the proposed classification can only be considered as premature. It remains to be demonstrated if fragments derived from growth hormone and placental lactogen are generated *in vivo*, exert antiangiogenic effects and can consequently be classified as vasoinhibins. Also, there is neither understanding as to which differences in molecular mass impact function of vasoinhibins nor is there any evidence of a clinical relevance of vasoinhibins derived from growth hormone and placental lactogen. Lastly, significant differences in sequence, structure, and function of prolactin, growth hormone, and placental lactogen exist between species and naturally, the smallest fraction of the total body of evidence derives from studies in humans. Accordingly, a sustainable classification that provides orientation in future research and in the clinical context can only be proposed on the basis of substantial information on the structure and biological function or dysfunction of vasoinhibins in human health and disease. Further, a classification of hormones of the vasoinhibin-family should be proposed after being subject of a consensus conference of experts in the field. We suggest that an appropriate occasion for this consensus conference are the FASEB-Conferences on “The Growth Hormone/Prolactin Family in Biology and Disease,” during which new evidence can be reviewed and the possibility of a sustainable classification of hormones of the vasoinhibin-family can be reevaluated. Until such agreement is met, the field will benefit from the precise description of each vasoinhibin tested and discovered.

## Conflict of Interest Statement

The authors declare that the research was conducted in the absence of any commercial or financial relationships that could be construed as a potential conflict of interest.
